# CKD-602, a topoisomerase I inhibitor, induces apoptosis and cell-cycle arrest and inhibits invasion in cervical cancer

**DOI:** 10.1186/s10020-019-0089-y

**Published:** 2019-05-28

**Authors:** Sungha Lee, Jung Yoon Ho, Jing Jing Liu, Hyewon Lee, Jae Young Park, Minwha Baik, Minji Ko, Seon Ui Lee, Youn Jin Choi, Soo Young Hur

**Affiliations:** 1 Departments of Obstetrics and gynecology, Gangseo MizMedi, Ganseogu Gangseoro 295, Seoul, 07639 Republic of Korea; 2 Department of Obstetrics and gynecology, Seoul St. Mary’s Hospital, College of Medicine, The Catholic University of Korea, 222, Banpo-daero, Seocho-gu, Seoul 06591 Seoul, Republic of Korea; 3 Cancer Research Institute, College of Medicine, The Catholic University of Korea, 222, Banpo-daero, Seocho-gu, Seoul 06591 Seoul, Republic of Korea; 4 Department of Health Sciences and Technology, SAIHST, Sungkyunkwan University, Gangnam gu Ilwonro 81, Seoul, 06351 Republic of Korea

**Keywords:** CKD-602, Cell cycle arrest, Cervical cancer, Topoisomerase inhibitor, Invasion assay

## Abstract

**Background:**

Cervical cancer is the third most common gynecological malignancy. Conventional treatment options are known to be ineffective for the majority of patients with advanced or recurrent cervical cancer. Therefore, novel therapeutic agents for cervical cancer are necessary. In this study, the effects of CKD-602 in cervical cancer were investigated.

**Methods:**

Three established human, immortalized, cervical cancer cell lines (CaSki, HeLa and SiHa) were used in this study. Following treatment with CKD-602, apoptosis was quantified using fluorescein isothiocyanate Annexin V-FITC and propidium iodide (PI) detection kit and cell cycle analysis was analyzed using fluorescence activated cell sorting (FACS). Transwell chambers were used for invasion assays. Western blot assay was performed to analyze proteomics. CaSki cells were subcutaneously injected into BALB/c-nude mice and cervical cancer xenograft model was established to elucidate the antitumor effect of CKD-602 in vivo.

**Results:**

Treatment with CKD-602 induced apoptosis and increased expression of the enzyme PARP, cleaved PARP, and BAX. In addition, expression of phosphorylated p53 increased. Cell cycle arrest at G2/M phase and inhibition of invasion were detected after treatment with CKD-602. A significant decrease in cervical cancer tumor volume was observed in this in vivo model, following treatment with CKD-602.

**Conclusions:**

This is the first report of CKD-602 having an antitumor effect in cervical cancer in both an in vitro and in vivo models. The results of this study indicate that CKD-602 may be a novel potential drug, targeting cervical cancer, providing new opportunities in the development of new therapeutic strategies.

**Electronic supplementary material:**

The online version of this article (10.1186/s10020-019-0089-y) contains supplementary material, which is available to authorized users.

## Background

Cervical cancer is the third most common gynecological malignancy and is responsible for 10–15% of cancer-related death in women (Jemal et al., 2011). Conventional treatment options (e.g., chemotherapy, radiation and surgical resection) are known to be ineffective for the majority of patients with advanced or recurrent cervical cancer, highlighting the need to identify novel treatment agents (Smith et al., 1993). Only modest response rates with excessive toxicity were reported without an improvement in survival rate (Chambers et al., 1994). Therefore, novel therapeutic agents for cervical cancer are necessary.

Type I and type II topoisomerase inhibitors interfere with DNA ‘unwinding’ during DNA replication and RNA transcription. Topoisomerase I (TOP1) cuts one strand in the double-stranded DNA, independent of ATP. In contrast, topoisomerase II (TOP2) cuts both strands in DNA and is dependent on **ATP** for its activity (Xu and Her**,** 2015**)**. Camptothecin (CPT) is the TOP1 inhibitor which was originally isolated from the Chinese tree *Camptotheca acuminate*, however it is no longer in use due to detrimental side effects It was replaced by more effective and safer TOP1 inhibitors such as topotecan and irinotecan (Wall and Wani, 1995).

The synthetic water-solube camptothecin analogue, CKD-602, 7-[2-(*N*-isopropylamino) ethyl]-(20S)-camptothecin (belotecan, Chong Kun Dang Pharmaceutical Corporation, Seoul, Korea), has been shown to be effective in the treatment of various cancers (e.g. glioblastoma, colon and ovarian cancer). The antitumor activity of CKD-602 in cervical cancer has not yet been fully investigated (Lee et al., 1998). In this study, the effects of CKD-602 in cervical cancer cell lines both in vitro and in vivo were investigated.

## Materials and methods

### Cell lines and reagents

Three established human, immortalized, cervical cancer cell lines (CaSki, HeLa and SiHa) were used in this study. All three cell lines were integrated with high-risk Human papillomavirus (HPV); CaSki and Siha cell lines were integrated with HPV-16 and the HeLa cell line was integrated with HPV-18. All cell lines were maintained in Roswell Park Memorial Institute (RPMI) 1640, Eagle’s Minimum Essential Medium (MEM) and Dulbecco’s modified Eagle’s medium (DMEM) (Gibco/ThermoFisher Scientific, Waltham, MA, USA) supplemented with 10% fetal bovine serum (FBS, Gibco) and 1% penicillin and streptomycin (Gibco) at 37 °C in a 5% CO_2_ incubator. The CKD-602 was dissolved in distilled water with 50 mg D-mannitol (Sigma–Aldrich, St Louis, MO, USA), 0.06 mg at 5 mg/1 ml tartaric acid (Sigma-Aldrich), and stored as stock in aliquots at 4 °C. For in vitro use, final concentrations between 15 and 150 ng/ml CKD-602 were obtained by appropriate dilutions of the stock solution in each cell medium. For in vivo use, final concentrations of 25 mg/kg CKD-602 were obtained by appropriate dilutions of the stock solution with RPMI 1640 medium (Gibco). The CKD-602 was donated from Chong Kun Dang Pharmaceutical Corporation (Seoul, Korea).

### Apoptosis assay

Apoptosis was quantified using the fluorescein isothiocyanate annexin V-FITC and propidium iodide (PI) Apoptosis Detection Kit I (BD Pharmingen, La Jolla, CA, USA) according to the manufacturer’s instructions. Briefly, CKD-602-treated and non-treated 1 × 10^6^ cells were washed in ice-cold phosphate buffered saline (PBS), resuspended in 100 μL of binding buffer, and incubated with 5 μL of annexin V-FITC and 5 μL of PI for 15 min in a dark room, at room temperature, as per manufacturer’s guidelines. Flow cytometric analysis was immediately performed using a FACS Calibur flow cytometer (FACS Calibur; Becton Dickinson, Franklin Lakes, NJ, USA).

### Cell-cycle analysis

The cells at a density of 2 × 10^5^ cells/well in MEM/DMEM with 10% FBS were added to the wells of a 6-well plate and incubated overnight. Each cell was treated with 1/2 IC50 and IC50 CKD-602 for 48 h. Following treatment, cells were harvested by centrifugation at 1300 rpm for 3 min, washed twice in ice-cold PBS and fixed by incubating the cells overnight at − 20 °C with 70% ethanol. Cells were then washed with ice-cold PBS and resuspended in 500 μl PI/RNase staining solution (Invitrogen/ThermoFisher Scientific, Waltham, MA, USA). The cell cycle position was evaluated by FACS using an excitation laser set at 480 nm and a detection wavelength of 575 nm. A minimum of 10,000~30,000 events/sample was analyzed.

### Cell invasion assay

A cell invasion assay was performed to elucidate whether CKD-602 is capable of inhibiting cervical cancer cell invasion (in CaSki, Hela and Siha cell lines). Transwell chambers were used (Corning INc., Corning, NY, USA) and 100 ul cells (1-2 × 105 per well) added to the upper chamber of 24-well plates, which was pre-coated with 30 μl of Matrigel (R&D Systems. catalog no. 1918-FN) for 2 h under a cell incubator. Concurrently, CKD-602 was diluted with 0.65 ml 20% FBS-DMEM complete medium(final concentration for CaSki, HeLa and Siha were: 120 ng/ml; 300 ng/ml and 300 ng/ml, respectively)was added to the lower chamber. After 48 h, the invaded cells in the lower chamber were fixed, stained with 0.1% crystal violet solution and then photographed under inverted microscope (× 200).

### Western blot analysis

Briefly, CKD-602-treated and non-treated cells were washed twice in PBS and cell lysates prepared using the radioimmunoprecipitation assay (RIPA). For immnunoblotting, 30 μg protein was resolved on 4–12% SDS-PAGE, transferred onto PVDF membrane (Bio-Rad, Hercules, CA, USA) by electroblotting, and blocked in 5% BSA in TBST. Membranes were then probed overnight at 4 °C with the following antibodies: Cyclin B1 (4138); cdc2 (9116); phopho-cdc2 (Tyr15; 9111); p21 (Waf1/Cip1;2947); p53 (2527); phopho-p53 (Ser15;9286), and PARP (9542) (1:1000; Cell Signaling Technology, Inc. (Danvers, MA, USA). Antibodies to c-PARP (ab32064; 1:1000), phopho-Cyclin B1 (S126; ab133439; 1:10,000), BCL2-associated X protein (Bax, ab32503; 1:2000), matrix mettloproteinases 2 (MMP2, ab97779; 1:5000), and vascular endothelial growth factor (VEGF, ab52917; 1:5000) and Cyclin A1 (sc56301; 1:1000) were acquired from Abcam (Cambridge, UK). The antibodies for CDK2 (sc6248; 1:5000) and the housekeeping gene, glyceraldehyde 3-phosphate dehydrogenase (GAPDH, sc32232, 1:5000) were acquired from Santa Cruz Biotechnology (Dallas, TX, USA).

The cell membrane was washed with phosphate buffered saline with Tween 20 (PBST) and incubated with corresponding secondary antibodies for 1 h at room temperature. Membranes were washed again with PBST and blots were developed into X-Ray films using the enhanced chemiluminescence (ECL) method previously described, or the Chemidoc MP system, Bio-Rad.

### Xenograft model

All animal experimental protocols were reviewed and approved by the Catholic University Animal Care and Use Committee and performed in accordance with the National Institute of Health Guide for the Care and Use of Experimental Animals. Normal six-week-old female BALB/c-nude mice (17–22 g) were purchased from Orient Bio (Gyeonggi-do, Korea). Specifically, CaSki cells (4 × 10^6^/head) were subcutaneously injected into the right leg of the mouse. After two weeks, when the tumor had attained an average volume of approximately 80mm^3^, tumor sizes were measured using a vernier caliper weekly and tumor volume calculated [(tumor length) × (tumor width) ^2^/2]. The dose of CKD-602 (25 mg/kg) was chosen based upon previous reports (Kim et al., 2013). The CKD-602 was injected intravenously for 16 days at 4-day intervals and PBS as a negative control was injected in same way. After CKD-602 treatment, tumor volumes and body weights were measured every 3–4 days from day 17. Mice were killed 4 weeks after establishment of the xenograft.

### Statistical analysis

All statistical analyses were performed using one-way ANOVA comparing variable groups (Graphpad Prism5 software). All experiments were repeated at least three times. In certain cases, the Student’s t-test was also performed. Values are expressed as mean ± standard error of the mean (SEM) for control and treated samples. A *p* value of < 0.05 was considered statistically significant (*p < 0.05, **p < 0.01, ***p < 0.001, ns = not significant).

## Results

### CKD-602 promotes pro-apoptotic activity in cervical cancer

**Fig. 1:**
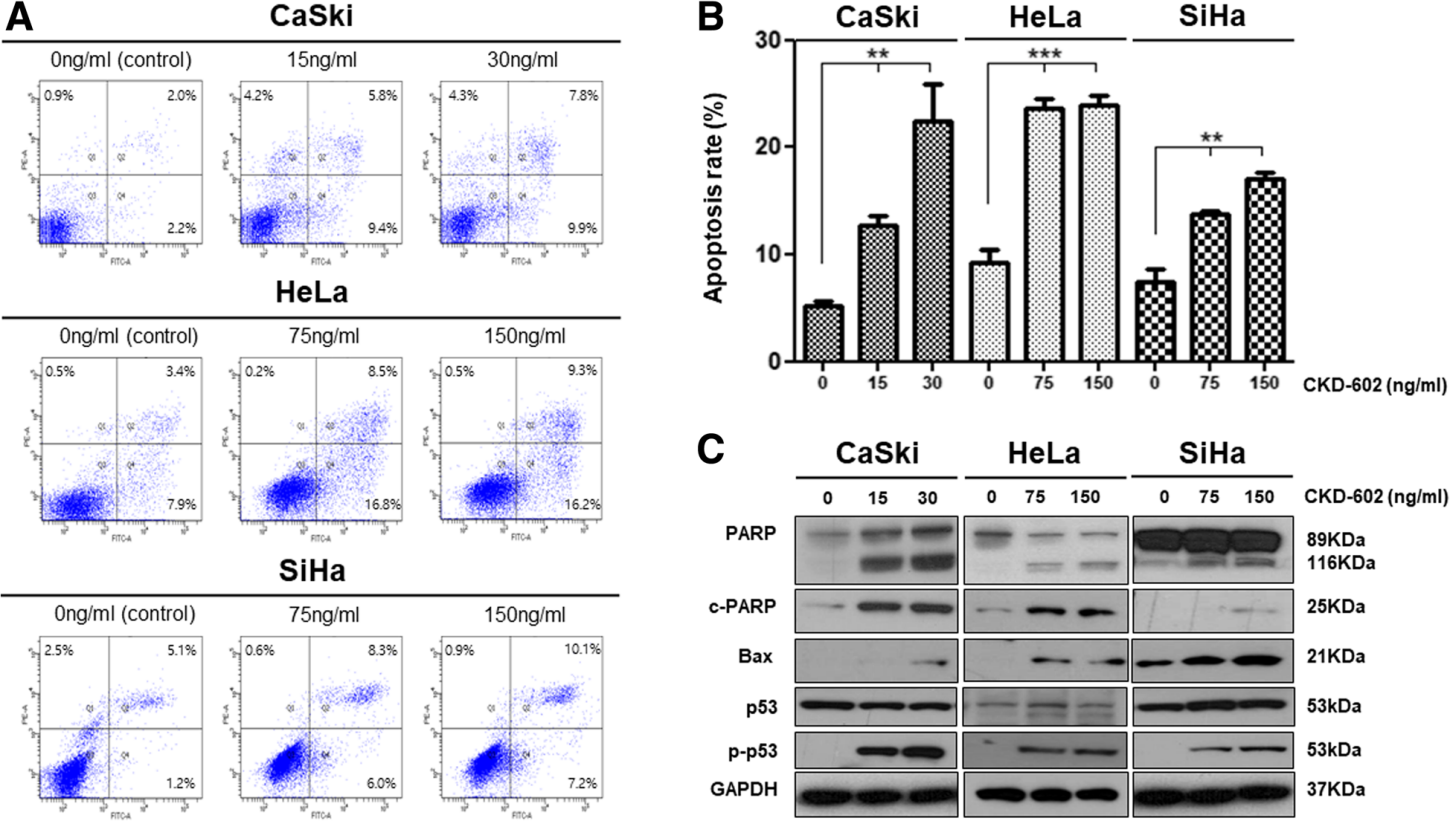
Pro-apoptotic activity in cervical cancer cells (CaSki, HeLa and SiHa) following CKD-602 treatment. Cells were incubated with CKD602 in a dose-dependent manner (0 [control], ½ IC50, IC50 ng/ml each cells) for 48 h. **a.** The percentage of apoptosic cells was evaluated using flow cytometic analysis with Annexin V and PI staining. **b.** The percentage of apoptosic cells measured by flow cytometry. Results are expressed as the mean ± SEM (*p < 0.05 and **p < 0.01). **c.** Western blot analysis of apoptosis-related proteins in cervical cancer cells

Using the cell viability assay, treatment with CKD-602 showed a significant cytotoxic effect in all cervical cancer cell lines in a time- (*p* < 0.05) and dose- (*p* < 0.05) dependent manner (Additional file 1: Figure S1). The IC50 (50% inhibition concentration of cell viability) values were 30 ng/ml (95% CI: 18.29–63.30) for Caski cells, 150 ng/ml (95% CI: 100.3–179.4) for HeLa cells, and 150 ng/ml (95% CI: 64.63–254.3) for SiHa cells at 48 h after treatment. To investigate the efficacy of CKD-602 in cervical cancer, pro-apoptotic activity was measured. A strong pro-apoptotic activity was observed in the treatment groups after 48 h of treatment (Fig. 1a and b). Compared to the control, apoptosis rates significantly increased in Caski, HeLa and Siha when treated with different concentrations (half the IC50 and IC50 values). The treatment increased the expression of PARP, cleaved PARP and Bcl2-associated X protein (BAX). In addition, expression of both p53 and phosphorylated p53 (Ser15) was increased (Fig. 1c).

### CKD-602 induces cell-cycle arrest in the G2/M phase in cervical cancer

**Fig. 2:**
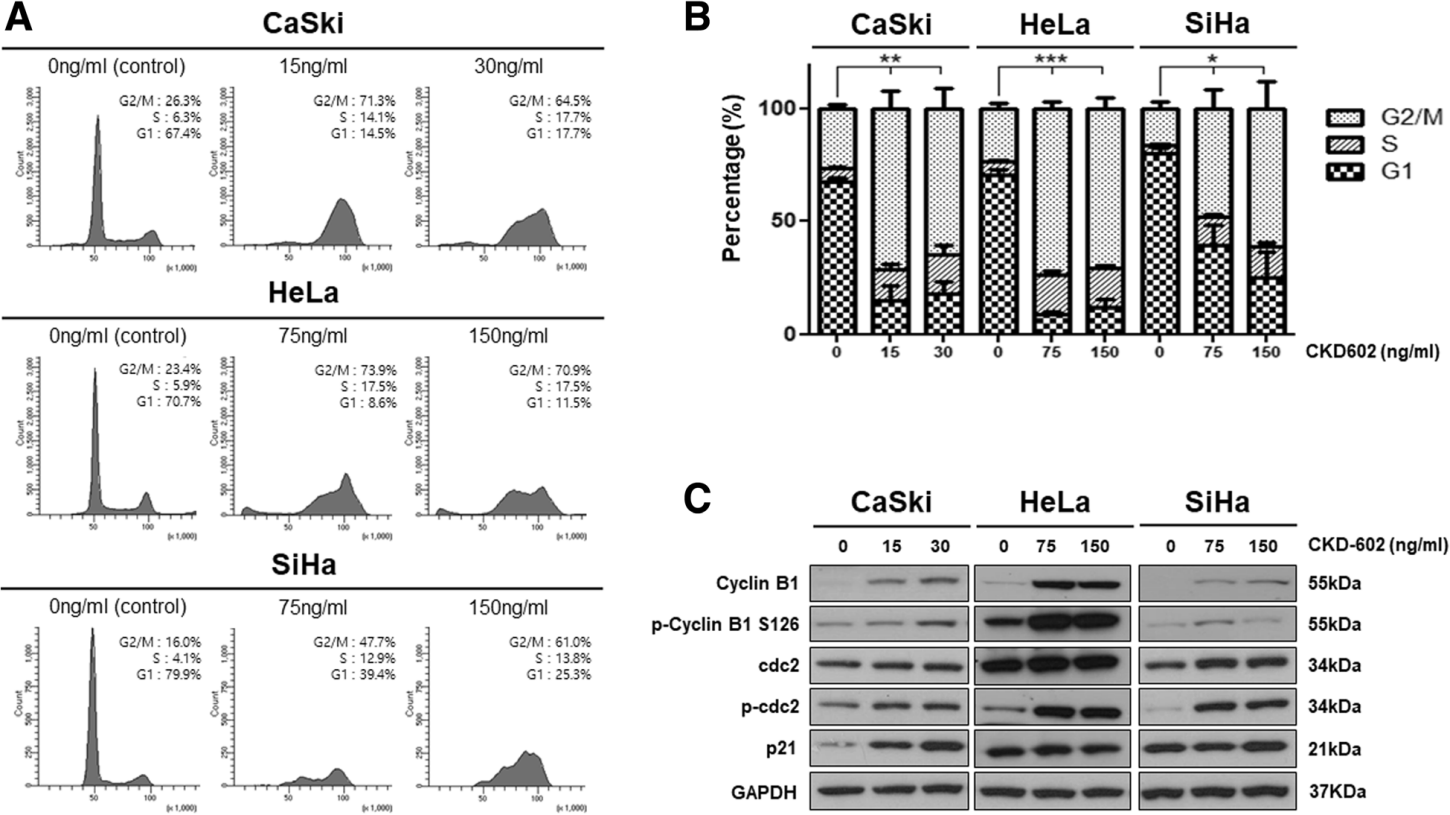
Induction of G2/M arrest in cervical cancer cells following treatment with CKD-602. **a.** Cells were incubated with CKD602 in a dose-dependent manner (0, ½ IC50, IC50 ng/ml each cells) for 48 h. Cells were then harvested and fixed with ethanol followed by PI staining to determine cellular distribution in different phases of the cell cycle using flow cytometry. **b.** Percentage of cell cycle progression measured by flow cytometry. Results are expressed as the mean ± SEM (*p < 0.05 and **p < 0.01). **c.** Western blot analysis of cell cycle-related proteins in cervical cancer cells

Cell cycle analysis was performed following CKD-602 treatment with different concentrations (half the IC50 and IC50 values) after 48 h of treatment (Fig. 2). Treatment induced the G2/M phase cell accumulation in all cell lines in a concentration-dependent manner. The proportion of the cell population in G2/M phase at 48 h increased from 26.3 to 64.5% (CaSki cells), 23.4 to 70.9% (HeLa cells) and 16.0 to 61.0% (SiHa cells). This increase was statistically significant (*p < 0.05 and ** *p* < 0.01). We investigated the G2/M – related protein levels. The increases in cyclin B1 and phosphorylated cyclin B1 (phosphor Ser126) expression levels were present in all the cell lines. Expression of Cdc2 protein did not show substantial change but phospho-cdc2 (Tyr15) protein expression increased after CKD-602 treatment.

### CKD-602 inhibits cancer invasion in cervical cancer

**Fig. 3:**
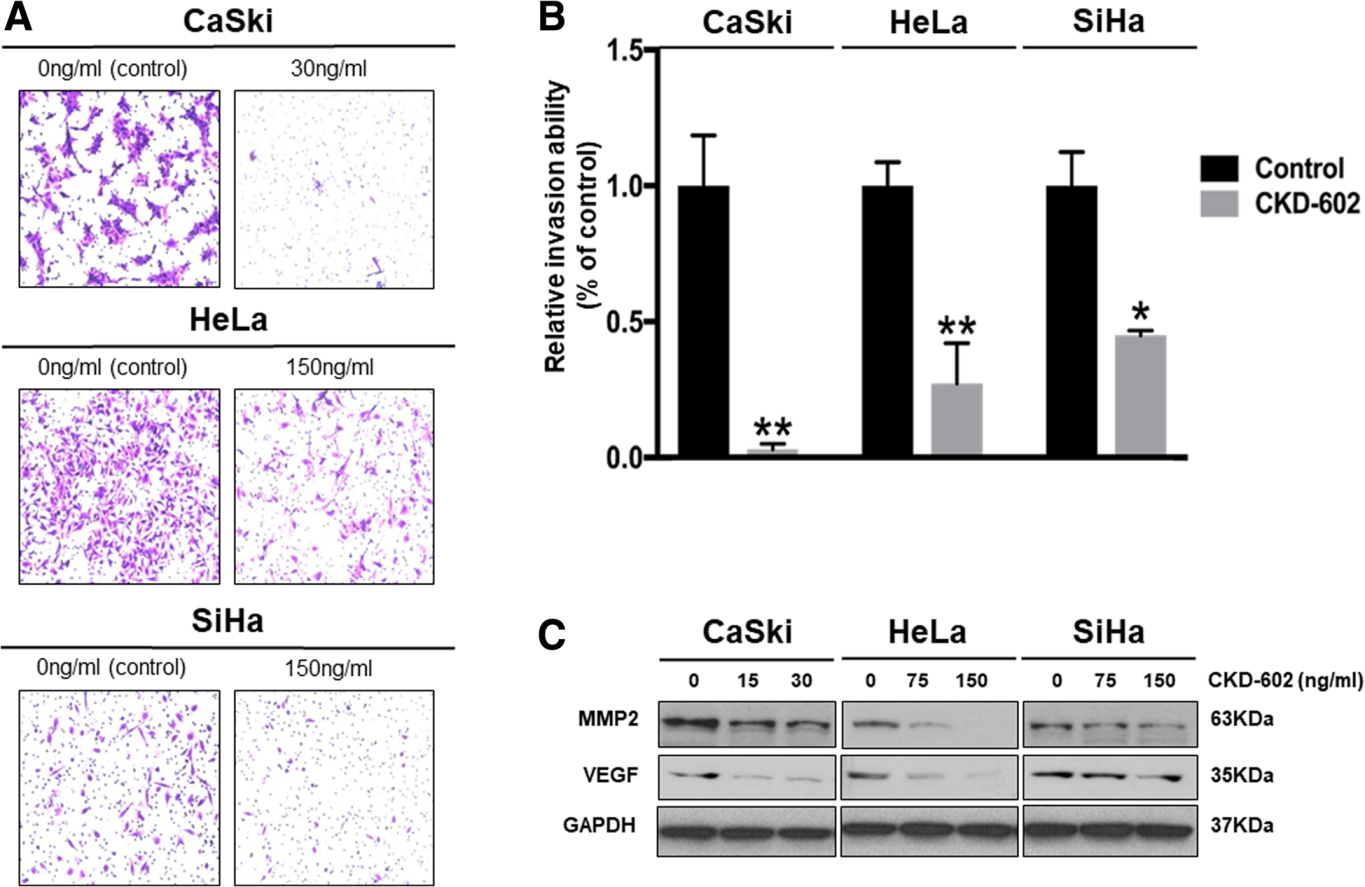
Invasive ability of CKD-602 in cervical cancer cells. **a.** Photomicrographs showing invasive potential of CKD602 in cervical cancer cells using matrigel precoated transwell assay (× 200) and **b.** Mean ± SEM (*p < 0.05 and ** *p* < 0.01). **c.** Western blot analysis of cell invasion-related proteins in cervical cancer cells

An in vitro invasion assay was performed to investigate whether CKD-602 treatment affected the invasive ability of cervical cancer cells. The assay was performed in chambers that had the upper wells coated with Matrigel to mimic the extracellular matrix in vivo. All the cervical cancer cell lines were treated with IC50 concentrations and representative photomicrographs of the membrane-associated cells (× 200) taken and analyzed (Fig. 3a). Significant differences of invasive ability were detected between the control cells and the CKD-602 treated cells (Fig. 3b) (*p < 0.05 and **p < 0.01). The protein expression levels of MMP2 and VEGF, two proteins known to be specifically related to the invasive ability of cells, decreased following CKD-602 treatment (Fig. 3c).

### Inhibition of tumor growth in CaSki-xenografted nude mice

**Fig. 4:**
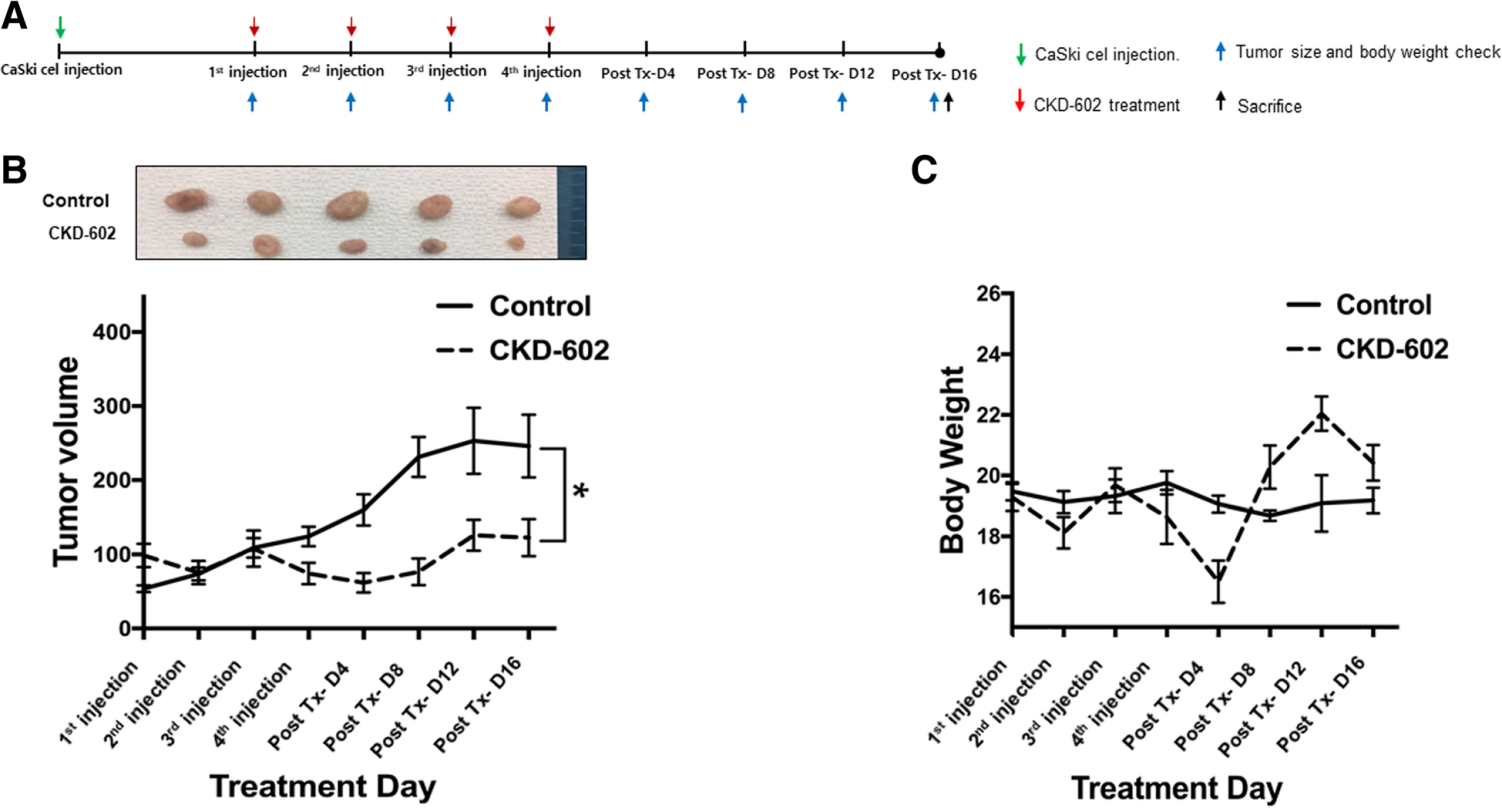
Antitumor efficacy of CKD-602 in a CaSKi xenograft model. **a.** Schematic diagram of the treatment regimen of CKD-602. Groups of BALB/c-nude mice (five per group) were subcutaneously challenged with CaSki 4 × 10^6^ CaSki tumor cells per mouse. **b.** Tumor volume in CaSki tumor-bearing mice in control mice and the CKD-602-treated mice. Results are expressed as mean ± SEM (*p < 0.05). **c.** Body weight of CaSki tumor-bearing mice in control mice and CKD-602-treated mice

Based on the in vitro data, it was then investigated whether CKD-602 would also be effective in an in vivo model. Groups of BALB/c-nude mice (five per group) were subcutaneously injected with CaSki cells and then treated (intravenously) with CKD-602 (25 m/kg) or PBS two weeks after the transplantation, when the tumors were approximately 80 mm^3^ (Fig. 4a). Seventeen days after the last injections, the mice were killed and the tumor volume measured and body weights of the mice recorded (Fig. 4b). Treatment with CKD-602 significantly inhibited the tumor growth of in this xenograft model compared with the control (*p* < 0.05). There was no significant difference in bodyweight between the xenograft mice and the controls, indicating that treatment with CKD-602 was well tolerated (Fig. 4b).

## Discussion

Topoisomerase I (TOP1) inhibitors (e.g. toptecan and iritonecan) are currently used in the treatment of cervical cancer treatment (https://www.nccn.org). Previous studies have demonstrated that CKD-602, a TOP1 inhibitor, is effective in the treatment of various cancers such as small cell lung carcinoma, oral squamous cell carcinoma and glioblastoma (Kim et al., 2013; Kim et al., 2015; Lee et al., 1998). In the current study, treatment with CKD-602 as a potential anticancer drug targeting cervical cancer was investigated for the first time. In both in vitro and in vivo models, treatment with CKD-602 promoted apoptosis, induced cell cycle arrest in G2/M phase and inhibited cancer invasion.

Based upon previous reports indicating that CKD-602 has anticancer effects (Kim et al., 2013; Lee et al., 1998) a possible pro-apoptotic effect in cervical cancer was investigated. Using three distinct human cervical cancer cell lines (CaSki [HPV − 16], HeLa [HPV-18], and SiHa [HPV-16]), it was demonstrated that activation of BAX and PARP cleavage were enhanced 48 h after treatment (Fig. 1). In addition, phosphorylation of p53 occurred after treatment. Previous studies have reported that phosphorylation of p53 modulates the functions of p53 and is causally related to apoptosis (Milne et al., 1995; Zhang et al., 1994). Furthermore, it has been demonstrated that p53 can activate the expression of BAX, a member of the Bcl-2 family, and that BAX can act to accelerate apoptosis (Basu and Haldar, 1998; Haupt et al., 2003; Miyashita et al., 1994). Pro-apoptotic signaling induced by TOP1 inhibitors has been reported in previous studies, suggesting that TOP1 possesses a protein-kinase activity that is specific to serine residues (García et al., 2014; Gobert et al., 1999; Tazi et al., 1997). Together, the data from the current study indicates that treatment with CKD-602 may induce apoptosis through phosphorylation of p53 in cervical cancer.

The effect of CKD-602 treatment in cell-cycle arrest in cervical cancer was investigated. It was found that treatment with CKD-602 is capable of inducing G2/M phase cell-cycle arrest in cervical cancer. Cell-cycle arrest at the G2/M phase is associated with enhanced apoptosis and is a cellular response to various DNA-damaging agents (DiPaola, 2002). A previous study has demonstrated CKD-602-induced cell-cycle arrest at the G2/M phase in oral squamous cell carcinoma cancer and the data from the current study indicates that the same may be true in cervical cancer (Kim et al., 2015). As shown in Fig. 2, expression of cyclin B1, phosphorylated cyclin B1 and p21 increased in cervical cancer following CKD-602 treatment. It is well known that p53 regulates the G2/M checkpoint through cyclin B1 (Innocente et al., 1999) however it has also been reported that some TOP1 inhibitors can induce p53-independent G2/M arrest (Bozko et al., 2002; Wu et al., 2015). Further studies are required to determine if G2/M arrest after CKD-602 treatment is p53-dependent or independent.

Several TOP1inhibitors have been reported to be involved in the inhibition of cancer invasion (Elstner et al., 2007; Min et al., 2011). The results of the current study are the first to show that inhibition of cancer invasion by CKD-602, a TOP1 inhibitor. It was found that expression of MMP2 and VEGF decreased 48 h after CKD-602 treatment in 3 cervical cancer cell lines (CaSki, HeLa and SiHa). Matrix metalloproteinases (MMPs), a family of enzymes, proteolytically degrade various components of the extracellular matrix (ECM). Increased expression of MMPs is associated with the increase of tumor cells in an organ (Zhang et al., 2015).Vascular epithelial growth factor (VEGF) is a mitogen that induces a rapid and complete angiogenic response to cancersous/tumor cells in an organ (Carmeliet, 2005). In addition, a previous study has shown that cell invasion is promoted via VEGF and MMP2 in cervical cancer (Chen et al., 2014).

## Conclusions

This is the first study to identify and report the anticancer effects of CKD-602 treatment in cervical cancer, in both in vitro and in vivo models. The data suggests that p53-mediated apoptosis, cell cycle arrest at G2/ M phase and inhibition of invasion through MMP2 and VEGF were involved in these processes. CKD-602 may be a potential drug targeting cervical cancer that will lead to new opportunities to develop new therapeutic strategies.

## Additional file

Additional file 1: **Figure S1**. Cell viability following CKD-602 treatment. (TIF 130 kb)

## Abbreviations

BAX: Bcl2-associated X protein
CPT: camptothecin
FACS: fluorescence activated cell sorting
MEM: Minimum Essential Medium
PI: propidium iodide
RPMI: Roswell Park Memorial Institute
TOP1: topoisomerase I
TOP2: topoisomerase II
