# Supplementary figures and images for: CKD-602, a topoisomerase I inhibitor, induces apoptosis and cell-cycle arrest and inhibits invasion in cervical cancer
Source: Mol Med. 2019 May 28;25:23. doi: 10.1186/s10020-019-0089-y (PMC6540464; doi:10.1186/s10020-019-0089-y)

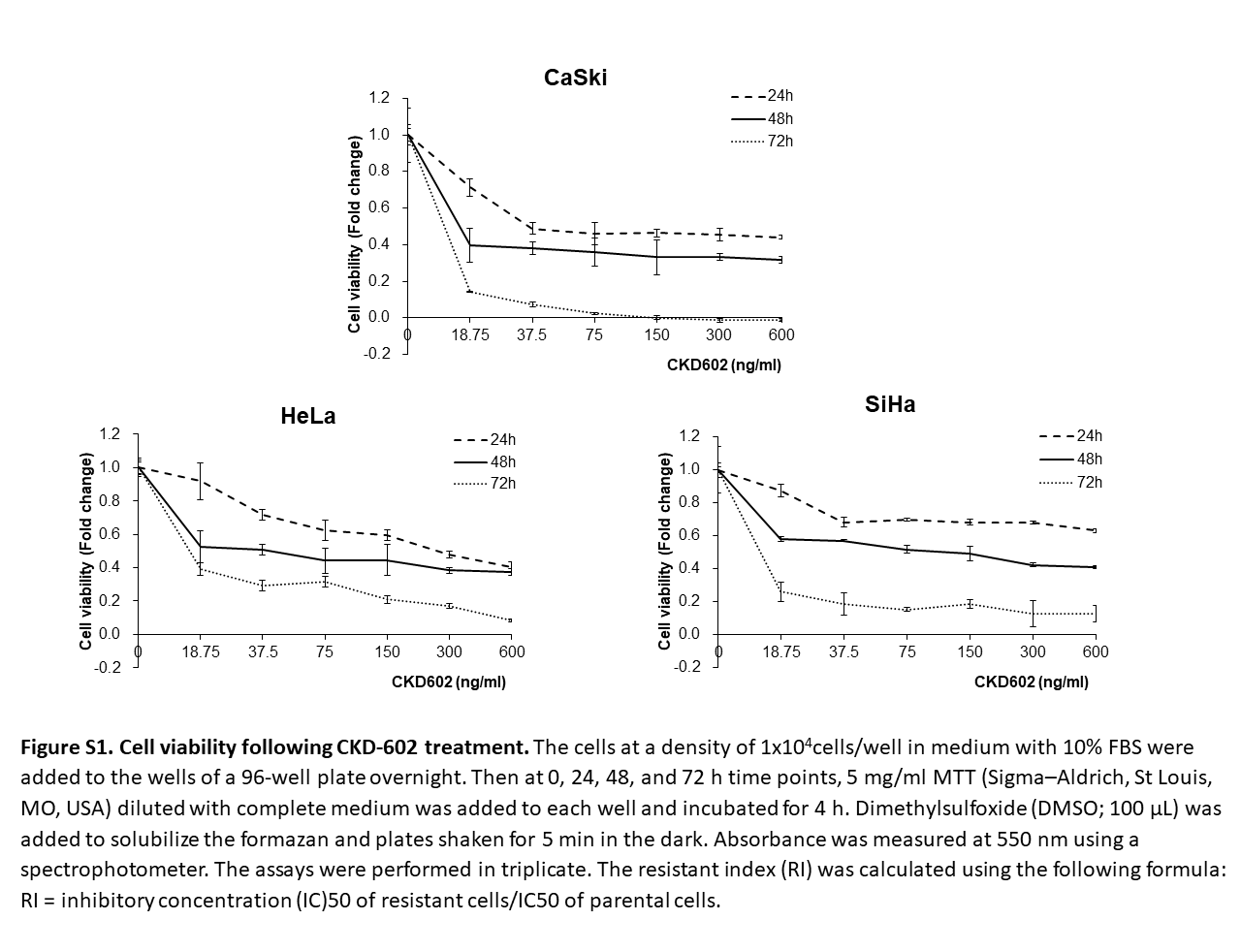

Supplement: Supplementary file 1 — Figure S1. Cell viability following CKD-602 treatment. (TIF 130 kb) [file 10020_2019_89_MOESM1_ESM.tif]
